# Feeder-Cell-Free and Serum-Free Expansion of Natural Killer Cells Using Cloudz Microspheres, G-Rex6M, and Human Platelet Lysate

**DOI:** 10.3389/fimmu.2022.803380

**Published:** 2022-03-07

**Authors:** Christopher D. L. Johnson, Nicole E. Zale, Eric D. Frary, Joseph A. Lomakin

**Affiliations:** Bio-Techne, Woburn, MA, United States

**Keywords:** natural killer (NK) cell, human platelet lysate (hPL), G-Rex6M, Cloudz microspheres, feeder-cell-free, xeno-free

## Abstract

The versatility of natural killer cells has ignited growing interest in their therapeutic use for cancer and other immunotherapy treatments. However, NK cells compose a small portion of peripheral blood mononuclear cells (5%–20% of PBMCs) and clinical doses require billions of cells. Manufacturing suitable doses of NK cells remains a major challenge for NK immunotherapy. The current standard for expanding NK cells relies on feeder cells and fetal bovine serum to achieve large expansion, but both encounter regulatory concerns. We developed NK Cloudz, a dissolvable polymer-based microsphere platform, as an alternative to a feeder cell approach to expand NK cells. We demonstrated that a combination of NK Cloudz, a G-Rex6M culture vessel, and GMP Human Platelet Lysate expanded NK cells 387 ± 100-fold in 10 days from a PBMC starting population. The NK purity, viability, and cytotoxicity were similar to both a feeder cell protocol and an FBS-based protocol. Additionally, we found no significant differences between FBS and GMP Human Platelet Lysate and concluded that platelet lysate is a good xeno-free alternative to FBS for NK expansion. Overall, we demonstrated a feeder-cell-free and FBS-free protocol that leverages NK Cloudz as a promising step toward a commercial GMP manufacturing method to expand NK cells for therapeutic use.

## 1 Introduction

Natural killer (NK) cells have received increasing attention as an allogenic cell therapy tool for cancer (1–5) and treatment of viral infections (6, 7) largely due to their capacity to recognize a variety of signals, like antibody-tagged targets, missing-self ligands, and stress-induced ligands (5, 8, 9). However, effective expansion of cytotoxic NK cells under good manufacturing practice (GMP) conditions to support commercial manufacture remains a challenge (6, 10, 11), as reviewed by Fang et al. (12).

Irradiated feeder cells are currently the standard for expanding NK cells *in vitro* (6, 11, 13–16). The K562 leukemia cell line has been modified to express ligands associated with antigen presenting cells (CD64, CD86, and truncated CD19) along with 4-1BB Ligand (CD137L) and membrane-bound IL-21 (K562-mbIL21-41BBL). The irradiated K562-mbIL21-41BBL have been shown to expand NK cells 47,967-fold in 21 days in RPMI media with 10% FBS (6). However, feeder cells can be complex to license, difficult to source, and difficult to remove from culture. Incomplete irradiation of the feeder cell population can lead to teratomas *in vivo*, yet even with sufficient irradiation, it is difficult to remove feeder cells after an expansion is complete, which leaves cultures with genetic material and other leukemic cell line components that may be injected into a patient (10). The potential quality control and safety challenges associated with K562 feeder cells have prompted a search for alternatives in support of clinical NK cell production. To this end, researchers sought to achieve similar NK expansion using autologous cells, with some success (17–20). Overall, the risks and complexities of relying on irradiated cell lines for GMP compliant expansion of clinical doses of NK cells have motivated development of feeder-cell-free NK cell expansion approaches *ex vivo*.

Feeder-cell-free approaches currently fall short of expansion rates achieved with feeder cells but have successfully expanded NK cells with high doses of cytokines (21–24), magnetic beads (25–28), or plasma-membrane-derived particles (10). The NK Cloudz^™^ platform, used here, offers a unique feeder-cell-free approach that uses polymer microspheres functionalized with humanized anti-CD2 and anti-NKp46 antibodies. Humanized antibodies have the benefit of reducing nontarget immunogenicity in comparison with their murine counterparts (29). Importantly, the Cloudz hydrogel microspheres can be quickly dissolved with a biocompatible release buffer and removed from culture to obtain a cleaner cell product for downstream applications.

Another remaining manufacturing challenge is to obtain a purified NK population. NK cells derived from induced pluripotent stem cells (iPSC) (26, 30) or the NK-92 cell line (31, 32) are promising approaches because they both expand purified NK populations and remove the effect of large variation from donors. However, it is currently more common to expand NK cells from a peripheral blood mononuclear cell (PBMC) starting population. The challenge with selective NK expansion from PBMCs is achieving high purity (6). As a result, the majority of methods include a nontarget-cell depletion before or after expansion (14, 15, 20, 27, 33–35).

A further complication for commercial NK cell expansion has been the reliance on fetal bovine serum (FBS) as a supplement in the cell expansion media (6, 10, 15, 23). Growing interest in scalable and clinic-ready NK culture methods has encouraged the development of xeno-free workflows. Recent work to convert the K562-mbIL21-41BBL expansion method to a serum-free process found that a commercial immune cell serum replacement was an effective alternative to FBS (36). Others have reported successful expansion with human AB serum (14, 26, 34, 37) or autologous plasma (20, 33). More recently, Huang et al. reported successful expansion of NK cells using human platelet lysate as a supplement (25).

In the current work, we sought to develop a feeder-free and FBS-free scalable process by leveraging the dissolvable Cloudz microsphere platform, the scalable G-Rex6M^®^ culture method, humanized antibodies, and GMP Human Platelet Lysate for a xeno-free process. The human platelet lysate was compared with FBS, and the overall culture method was compared with the K562-mbIL21-41BBL feeder cell culture method.

## 2 Materials and Methods

### 2.1 Cloudz

Cloudz are commercially available, dissolvable, magnet-free hydrogel microspheres designed to activate and expand NK cells through proximal stimulation with humanized anti-CD2 and anti-NKp46 antibodies. The microspheres are composed of an alginate copolymer, gelled into spherical particles in the presence of divalent salt ions. Cloudz hydrogel microspheres rapidly dissolve when exposed to a paired release buffer that contains a chelating agent to enable simple and complete removal of Cloudz within a scalable cell culture process [Cloudz Human NK cell Expansion Kit (Humanized Antibodies), CLD005, Bio-Techne].

### 2.2 Cell Culture

Natural killer cells were expanded from PBMCs in 100 ml of media in a G-Rex6M for 10 days using the NK Cloudz conjugated with humanized antibodies.

#### 2.2.1 PBMC Isolation

PBMCs were isolated from Trima Cones (Innovative Blood Resources, Saint Paul, MN, USA) using a Ficoll-Paque gradient. Briefly, Trima Cones were washed with phosphate-buffered saline (PBS) (B30250, Bio-Techne, Minneapolis, MN, USA) to remove the plasma layer, then carefully layered on top of the Ficoll-Paque PLUS (17544202, Cytiva, Marlborough, MA, USA) and centrifuged for 30 min at 800×*g*. The PBMC layer was transferred to a separate tube and washed with PBS, before a 10-min incubation with red blood cell lysis buffer (FC002, Bio-Techne). The lysis was quenched with 0.5 M HEPES solution (15630-80, ThermoFisher, Waltham, MA, USA) and washed twice with PBS. The PBMC population was raised in Cryostor CS10 (210102, BioLife Solutions, Bothell, WA, USA) then aliquoted for freezing. The aliquots were arranged in a cryo-freezing container (5100-001, Nalgene, Rochester, NY, USA) which was placed in a −20°C freezer for 30 min, then transferred to a −80°C freezer overnight. The following morning, the frozen aliquots were transferred to a liquid nitrogen storage tank (CMR-2800, ThermoFisher) until use.

#### 2.2.2 Cell Culture in G-Rex6M With Cloudz

Media were prepared using GMP SCGM media (20802-500, CellGenix, Portsmouth, NH, USA) supplemented with either 10% v/v GMP PLUS Human Platelet Lysate (Plt Lys, PLSGB, Compass Biomedical, Hopkinton, MA, USA) or 10% v/v fetal bovine serum (FBS, S11550, Bio-Techne) and cytokines: 27 ng/ml IL-2 (202-GMP), 10 ng/ml IL-12 (219-IL/CF), 10 ng/mL IL-18 (9124-IL/CF), and 10 ng/ml IL-21(8879-IL/CF) (IL-2/12/18/21, all from Bio-Techne). The media was sterile filtered through a 0.22 µm filter unit (5690020, Nalgene). The filtered media was warmed in a 37°C waterbath prior to the start of the cell culture. At the start of the culture, PBMC aliquots were removed from the liquid nitrogen and placed in a 37°C waterbath (Isotemp 205, ThermoFisher, Waltham, MA, USA) until thawed, then washed twice in prewarmed media (prepared without cytokines): PBMCs were transferred to 15 ml of media and centrifuged at 300×*g* for 10 min (RT1, ThermoFisher) to pellet the cells. The supernatant was discarded, and the cells were raised in 10 ml fresh prewarmed cytokine-free media. A sample was taken for analysis, and the remainder of the cells were placed in 37°C, 5% CO_2_ incubator (3110, ThermoFisher) to rest during analysis.

Cell counts were determined by preparing a 1:10 dilution in media and were analyzed using a Novocyte Flow Cytometer 3000 (Agilent Technologies, Santa Clara, CA, USA). Separate samples were prepared for phenotype analysis: samples were washed with PBS then stained for viability using the LIVE/DEAD™ Fixable Yellow Dead Cell Stain Kit (L34967, Invitrogen, Waltham, MA, USA). Samples were washed with flow buffer (PBS supplemented with 1% BSA (BP9700-100, ThermoFisher)), then stained with CD3-AlexaFluor 405 (FAB100V, Bio-Techne), CD56-APC (318310, Biolegend, San Diego, CA, USA), CD45-PE-cy7 (557748, BD Biosciences, Franklin Lakes, NJ, USA), CD16-PerCP (302030, Biolegend), NKp46-BB515 (564536, BD), NKG2D-PE (557940, BD), and CD2-AlexaFluor750 (FAB18561S, Bio-Techne). Samples were washed in flow buffer and analyzed using the Novocyte Flow Cytometer 3000. Results were compensated using OneComp eBeads (01111142, ThermoFisher) and gates were based on unstained controls. NK cells were defined as CD45^+^CD3^−^CD56^+^. The counts were multiplied by the percentage of viable CD45^+^CD3^−^CD56^+^ population to calculate the NK population.

After characterization, 500,000 NK cells were seeded into 100 ml of prewarmed media with cytokines in a G-Rex6M plate (Wilson Wolf, Saint Paul, MN, USA). The Cloudz were vortexed for 30 s and 75 µl of The Cloudz from the Cloudz Human NK Cell Expansion Kit were vortexed for 30 s, then 75 ul of cloudz solution was added to each G-Rex6M well. The plates were placed in the 37 C, 5% CO_2_ incubator for 10 days. On days 7 and 10, the cultures were mixed using a 10 ml pipette (07200574, Corning, Corning, NY, USA) and a 0.5 ml sample was removed for analysis. The Cloudz in each sample were released by adding an equal volume of 1× release buffer (1:2 dilution, Bio-Techne) and mixing 10× with a pipette before proceeding with the analysis described in the previous paragraph.

#### 2.2.3 Release Buffer Biocompatibility

The release buffer was developed to remove the Cloudz with minimal effect on the cells. The effect of the release buffer on cell viability was tested by exposing the cells to release buffer for increasing periods of time. The cells were released as described in **Section 2.2.2** by adding an equal volume of 1× release buffer to each of the 3 samples collected from the wells cultured in SCGM with 10% human platelet lysate. Each parallel sample received a different treatment: The first group remained in the release buffer for 30 min, the second group for 60 min, and the third group for 120 min. After incubation, the samples were centrifuged at 300×*g* for 5 min to pellet the cells. The supernatant was discarded, the cells were raised in 1 ml of fresh media, transferred to a 24-well plate, and incubated for 3 days in the 37°C, 5% CO_2_ incubator. As a control, one group was not exposed to release buffer but was centrifuged and cultured in the same manner as the experimental groups. After 3 days, the samples were stained for viability as described in **Section 2.2.2**.

#### 2.2.4 Cell Culture in T75 Flasks With K562-mbIL21-4-1BBL Feeder Cells

Feeder cells have long been the standard for NK expansion, and irradiated K562-mbIL21-41BBL cells have been particularly effective at expanding NK cells (6). We used the protocol developed by Somanchi et al. (38) and Denman et al. (6) as a comparison. Briefly, media was prepared in RPMI-1640 (12633012, Gibco, Waltham, MA, USA) by adding 10% FBS, 1% Glutamax (35050-061, Gibco), and 10 ng/ml (approximately 50 IU/ml) IL-2. The media was sterile filtered using a 0.22 µm filter unit. A total of 5 million PBMCs were seeded into 40 ml of prewarmed RPMI media in a T75 flask (430641U, Corning). K562-41BBL-mbIL21 feeder cells were added at a ratio of 2:1 Feeder : PBMC. On days 3 and 5, one-half of the media volume was replaced, and the cytokines were refreshed. Also on day 7, the culture was split to maintain a concentration of 0.25 × 10^6^ cells/ml, and the media was refreshed. Feeder cells were refreshed at a 1:1 Feeder : PBMC cell ratio.

#### 2.2.5 Cytotoxicity Assay

The cytotoxicity protocol was adapted from Somanchi et al. (39). 4 days prior to the assay, K562 cells were thawed in a 37°C waterbath and washed using RPMI-1640, 10% FBS, and 1× Glutamax. K562 cells were plated in a T75 flask at a concentration of 250,000 cells/ml in the RPMI-1640, 10% FBS, and 1× Glutamax media and placed in the cell culture incubator. Every 3 days, the media was refreshed, and the cell concentration was returned to 250,000 cells/ml. On the day of the assay, the K562 cells were washed and counted using the NovoCyte flow cytometer and the concentration was adjusted to 1 × 10^6^ cells/ml in media. Calcein (4892-010-01, Bio-Techne) dissolved in dimethyl sulfoxide (DMSO, 3176, Bio-Techne) was added to the solution to reach a concentration of 2 µg/ml. The cells were incubated in the 2 µg/ml calcein solution for 30 min in the 37°C incubator. Following the incubation, cells were washed with fresh media to remove excess calcein and the cell concentration was returned to 1 × 10^6^ cells/ml.

For the expanded and characterized NK cells, the Cloudz were first released with an equal volume dilution in release buffer, followed by 10× mixing using a pipette. The NK cells were raised in an appropriate volume of the RPMI-1640, 10% FBS, and 1× Glutamax media such that 100 µl would achieve the correct effector:target (E:T) ratio. For a 1:1 E:T ratio, cells were diluted to 1 × 10^6^ NK cells/ml. To start the assay, 100 µl of calcein loaded K562 target cells were mixed with 100 µl of NK effector cells in a round-bottom 96-well plate (353910, Corning). Negative controls were prepared by seeding 3 wells with 100 µl of calcein-loaded K562 cells and 100 µl of media. Positive controls were prepared by seeding 3 wells with 100 µl of calcein-loaded K562 cells and 100 µl of media containing 2% Triton X-100 (8511, ThermoFisher) to lyse the target cells.

After a 4 h incubation, the samples were mixed 5× with a pipette to free trapped calcein in cellular debris. The samples were then centrifuged for 2 min at 400×*g* to pellet the cells. A total of 100 µl of the supernatant was carefully transferred to a flat-bottom black-walled 96-well plate (3603, Corning). The samples were analyzed using a plate reader (Synergy HTX, BioTech, Winooski, VT, USA) with an excitation wavelength of 485 nm and an emission wavelength of 530 nm. The cytotoxicity was determined by first subtracting the negative control from all samples (including the positive control) to remove the background noise. All experimental samples were then divided by the positive control to determine the percentage of the total calcein released.

#### 2.2.6 Analysis

Analysis was performed using the NovoExpress 1.5.0 Software and Microsoft Excel. The gating strategy is described in **Supplementary Figure 1**. The fold change was calculated by dividing the number of viable CD45^+^CD3^−^CD56^+^ cells at each time point by the number seeded on Day 0. PBMC donors were not vetted prior to culture. Donor variation is an important known but uncontrollable confounding variable in the results, so at least 3 donors were tested for each condition to model donor variation. Graphs represent the mean ± standard deviation from at least 3 separate donors unless otherwise mentioned.

## 3 Results

### 3.1 Cloudz Expansion of NK Cells in the G-Rex6M Using Human Platelet Lysate

Materials and culture conditions were chosen with the goal of working toward a GMP compatible xeno-free process of expanding NK cells. Cytokines and media were supplemented with human platelet lysate as a GMP compatible xeno-free alternative to FBS (25). The G-Rex culture vessel was chosen because of its scalability due to its gas permeable, liquid impermeable, membrane that allows cells to be cultured with fewer media exchanges compared with a traditional flask. The G-Rex6M provides 6-wells, each with a 10 cm^2^ culture area and a 100-ml column of media on top. In the following experiments, cells in the G-Rex6M were mixed on day 7 to provide an intermediate timepoint for the analysis. No media changes or other manipulation was performed.

**Figure 1:**
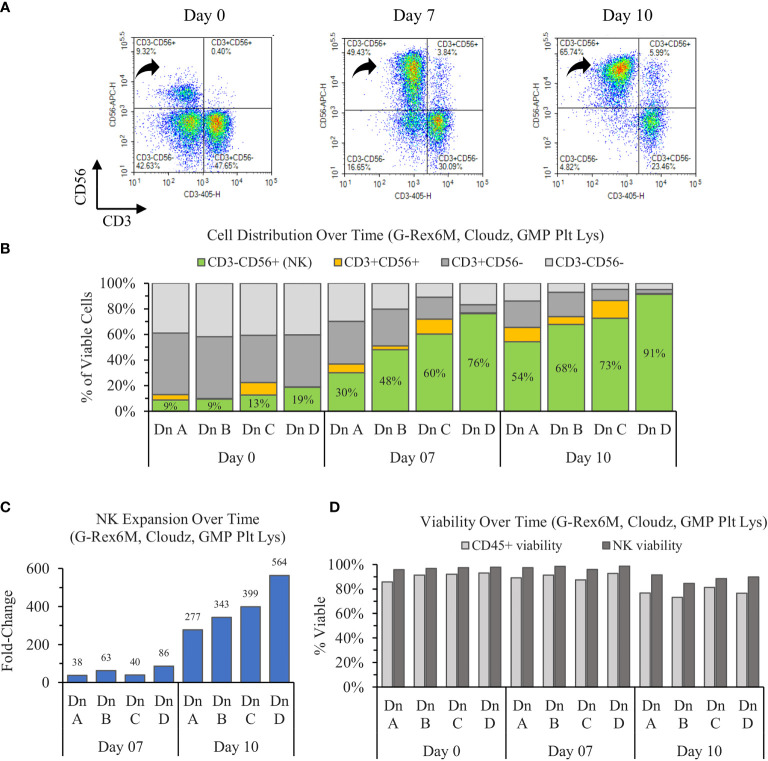
Ten-day expansion of NK cells from PBMCs with NK Cloudz and GMP human platelet lysate cultured in the G-Rex6M. **(A)** Flow cytometer density plots at 0, 7, and 10 days compare CD3 and CD56 expression of the expanded cell population. Black arrows indicate CD3^−^CD56^+^ (NK) cells, which correspond with the green boxes in **(B)** the plot of cell distribution of viable CD45^+^ cells. **(C)** Viable NK cell fold change relative to day 0. **(D)** The viability of two populations: the light gray bars represent the viability of all CD45^+^ cells (including NK cells) and the dark gray bars represent the viability of only the CD45^+^CD3^−^CD56^+^ (NK) population. The graphs are broken out by donor (Dn A, Dn B, Dn C, and Dn D) to highlight the donor variability in the process.

The results show that the initially CD56dim population of NK cells on day 0 enriches toward a largely CD56bright population on day 7, then consolidates to a population with a CD56 brightness in between the days 0 and 7 values (**Figure 1A**). When averaged, the three donors increase in purity from 12% ± 5% to 54% ± 19% to 71% ± 15% on days 0, 7, and 10, respectively. The NK enrichment was coupled with a proportional loss in CD3^+^CD56^−^ (T cells) and CD3^−^CD56^−^ (Other) CD45^+^ cells. However, the CD3^+^CD56^+^ (NKT cell) proportions remained relatively consistent over time within each donor (**Figure 1B**).

The starting population of 500,000 NK cells expanded 55 ± 26-fold by day 7 and 387 ± 100-fold by day 10. When the fold change over the time intervals was considered, the NK cells expanded 55 ± 26-fold between days 0 and 7 and 7 ± 2-fold between days 7 and day 10 (**Figure 1C**). While the number of cells increased 387-fold in 10 days, the rate of growth was highest in the first 7 days. The NK viability remained greater than 95% for the first 7 days, then decreased to 89% ± 3% on day 10. The NK viability remained 9% higher than the overall CD45^+^ population on average for all timepoints tested (**Figure 1D**), which suggests that the culture method favors NK cells. Overall, the cultures enriched from 12% ± 5% on day 0 to a purity of 71% ± 15% and a viability of 89% ± 3% by day 10. The NK cell population expanded 387-fold on average, which represents an average 198 ± 61 million viable NK cells (CD45^+^CD3^−^CD56^+^) in the G-Rex6M on day 10.

The variation between donors A and D prompted an analysis to determine if the differences in purity, expansion, or viability were correlated with phenotype markers on day 0. An analysis of both the percentage of the population and the MFI for CD56, CD16, NKp46, NKG2D, and CD2 revealed no relationship between donors and the outcomes. Furthermore, the percentage of alternate populations within the culture (CD3−CD56−, CD3+CD56+, and CD3+CD56−) were not able to predict the purity or expansion achieved by a donor.

### 3.2 Comparison of Human Platelet Lysate With Fetal Bovine Serum

**Figure 2:**
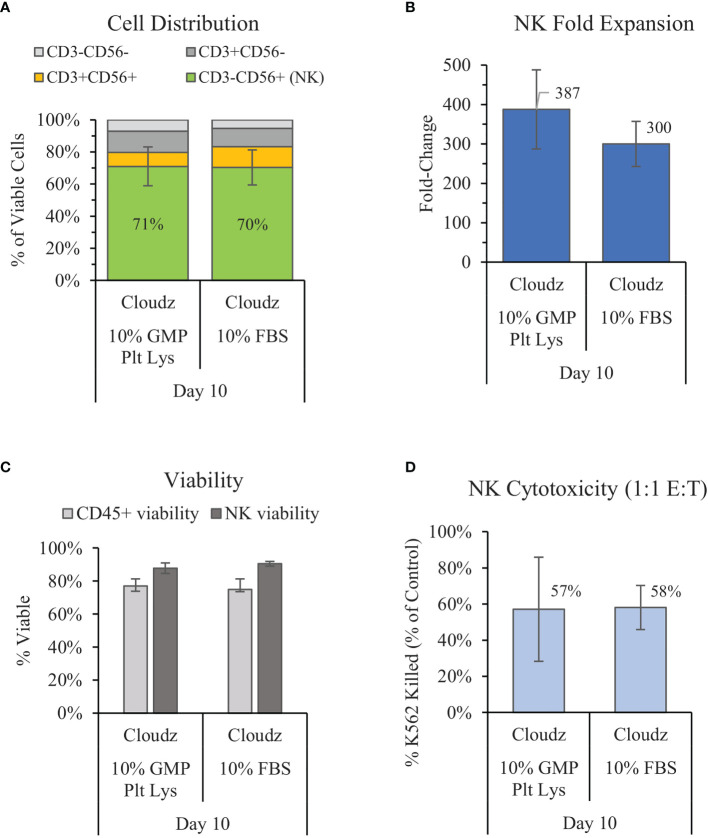
Comparison of the xeno-free 10% GMP human platelet lysate/NK Cloudz protocol to the existing FBS/NK Cloudz protocol in the G-Rex6M. The results show **(A)** the cell distribution on day 10, **(B)** NK (CD3^−^CD56^+^) fold change between days 0 and 10, **(C)** the viability of either all CD45^+^ cells or NK cells alone on day 10, and **(D)** the percent of target K562 cells killed on day 10 with a 1:1 effector:target (E:T) ratio and a 4 h incubation. Data represent the mean ± SD from 4 separate donors. The GMP human platelet lysate resulted in similar purity, expansion, viability, and cytotoxicity as the FBS protocol.

Previous work by Lapteva et al. has shown successful expansion of NK cells in the G-Rex6 platform (40 ml) using SCGM media with 10% FBS and K562-mb15-41BBL feeder cells (15). We compared a similar method with G-Rex6M (same culture area but 100 ml volume), feeder-cell-free NK Cloudz, and either 10% FBS or 10% GMP PLUS Human Platelet Lysate. The results show that substituting human platelet lysate for FBS resulted in a similar purity (71% vs. 70% for FBS) and viability (87% vs. 90% for FBS) but a higher mean fold-expansion (387 ± 100-fold vs. 300 ± 57-fold in FBS) (**Figures 2A–C**). The differences were not statistically significant. The expanded NK cell functionality was assessed with a cytotoxicity assay. The cytotoxicity assay measured calcein release from K562 cells after a 4 h incubation with a 1:1 E:T ratio. The results showed that the cells expanded using human platelet lysate had a similar cytotoxicity (57%) to those expanded with FBS (58%) (**Figure 2D**). These results validate GMP PLUS Human Platelet Lysate as an equivalent xeno-free alternative to FBS in our G-Rex6M culture method.

### 3.3 Comparison of Cloudz/G-Rex Low-Touch Method With the High-Touch Feeder Cell Method

**Figure 3:**
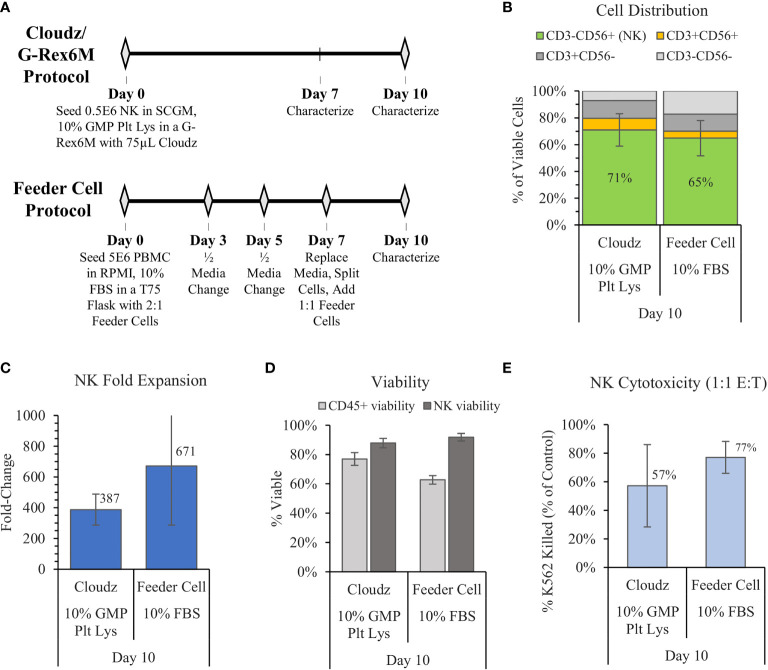
The G-Rex6M/NK Cloudz™ “low-touch” protocol compared with the flask-based “high-touch” feeder cell protocol. The feeder cell protocol used the same donors but was otherwise different: (See methods in **(A)** or in the Materials and Methods section). The results show the following: **(B)** the cell distribution on day 10, **(C)** the NK (CD3^−^CD56^+^) fold change between days 0 and 10, **(D)** the viability of either all CD45^+^ cells (light gray bars) or NK cells alone (dark gray bars) on day 10, and **(E)** the percent of target K562 cells killed with a 1:1 effector:target (E:T) ratio over 4 h. Data represent the mean ± SD from 3 separate donors.

In 2012, Denman et al. showed that feeder cells more effectively expanded NK cells when membrane-bound IL-15 was exchanged for membrane-bound IL-21 (6). Here, we used the Denman et al. protocol for K562-mbIL21-41BBL as a comparative control for the G-Rex6M expansion protocol with human platelet lysate and Cloudz. The two protocols differed significantly. The Denman et al. feeder cell protocol cultured cells in a T75 flask using RPMI, 1% Glutamax, 10% FBS, and 10 ng/ml (approximately 50 IU/ml) IL-2 media. Our protocol cultured cells in a G-Rex6M with SCGM, 10% GMP Human Platelet Lysate, 27 ng/ml IL-2, 10 ng/ml IL-12, 10 ng/ml IL-18, and 10 ng/ml IL-21 media. The Denman et al. protocol required more touchpoints, with media exchanges and cytokine addition every 2–3 days, along with a feeder cell replenishment on day 7 (**Figure 3A**). The Cloudz/G-Rex6M protocol resulted in a higher, but not statistically significant, mean NK purity (71% ± 12%) compared with the feeder cell protocol (65% ± 13%) (**Figure 3B**) and a similar viability (**Figure 3D**) on day 10. The feeder cell protocol resulted in nearly twice the mean fold expansion (671 ± 385-fold) compared with the Cloudz/G-Rex6M protocol (387 ± 100-fold); however, large variation between the donors in the feeder cell protocol meant that the difference is not statistically significant from the Cloudz/G-Rex6M protocol expansion (**Figure 3C**). A 4 h K562 cytotoxicity assay with a 1:1 E:T ratio showed that the feeder cell protocol resulted in a higher mean cytotoxicity (77% ± 11%) relative to the Cloudz/G-Rex6M protocol (57% ± 29%), although these differences were not statistically significant (**Figure 3E**). Our previous work using SCGM media with 10% FBS suggests that the differences observed in **Figure 3** are most closely related to differences in the protocol. When the K562-mbIL21-41BBL cells were cultured using the same protocol as the Cloudz, the mean expansion and cytotoxicity were similar to the results using Cloudz (**Supplementary Figure 2**).

Previous results suggest that the culture growth stagnates if the cell number reaches the carrying capacity of the G-Rex6M vessel (**Supplementary Figure 3**). We prepared an experiment to mimic the “high-touch” protocol using the Cloudz and the G-Rex6M vessel. Cultures were started in 8 ml in a G-Rex24 well plate. On day 7, the cultures were transferred to a G-Rex6M and raised to 100 ml with fresh media. On days 10 and 14, the cultures were analyzed, split, and 5,000,000 viable cells were transferred to a new well of a G-rex6M well plate. The results show that splitting the cultures was essential for maintaining the NK cell growth. On day 20 of culture, the mean NK purity was 96% ± 3%, the mean expansion was 55,106 ± 24,029, and the mean NK viability was 88% ± 3% (**Supplementary Figure 3**). The control, that was not transferred or split, peaked on day 10, then decreased in cell number over the remaining time, as expected.

Phenotype analysis was carried out by analyzing CD16, NKp46, NKG2D, CD2, TNF-a, IFN-g, IL-8, and Granzyme B. Analysis of the percentage of the NK population expressing CD16, NKp46, NKG2D, or CD2 for the FBS, human platelet lysate, and feeder cell groups resulted in no statistically significant differences between the groups (**Supplementary Figure 4**). The mean fluorescent intensity (MFI) shift between days 0 and 10 suggested that NKp46, CD2, and CD56 MFI increased by day 10, NKG2D MFI increased in 2 donors, and CD16 MFI decreased but remained positive (**Supplementary Figure 5A**). Pearson correlation tests revealed no consistent correlation between NK purity, fold change, and the changes in MFI of any markers measured (**Supplementary Figure 5B**). ELLA analysis of activating cytokines released into the media by day 10 showed similar expression of TNF-alpha, IFN-gamma, IL-8, and Granzyme B expression in the G-Rex6M/Cloudz cultures in either the FBS or human platelet lysate supplement. Interestingly, when the G-Rex6M/Cloudz/platelet lysate culture method was compared with the feeder cell protocol, the feeder cell protocol resulted in reduced IFN-gamma and IL-8 concentrations (**Supplementary Figure 6**).

**Figure 4:**
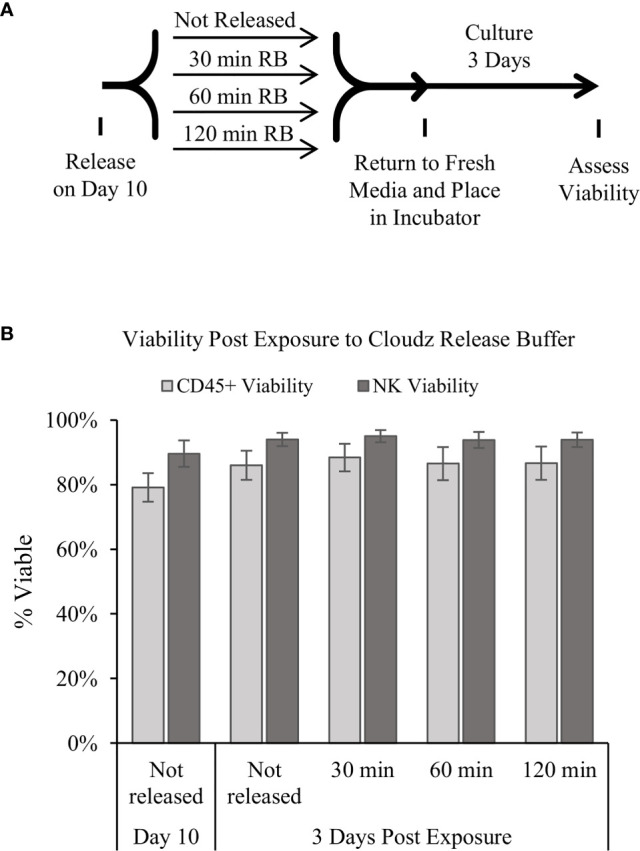
Release of the cells and extended culture. **(A)** Diagram of the protocol used to test how extended periods in the release buffer affect cells. Samples were mixed, and then an equal volume of 1× release buffer was added to the cells to release the Cloudz. Samples were incubated in this release buffer for either 30, 60, or 120 min, then washed, returned to growth media, and cultured for 3 days. **(B)** The viability of either all CD45^+^ cells (light gray bars) or NK cells alone (dark gray bars). Data represent the mean ± SD from 3 separate donors. The results suggest that extended incubation has no effect on viability compared with a sample that was not released.

Releasing the cells for downstream use was modeled by adding the release buffer then transferring the cells to a 24-well plate for further culture. The effect of the release buffer was tested by exposing the cells to release buffer for 3 periods of time: 30, 60, or 120 min. The 30-min exposure represents the longest time that we have observed during routine processing of the cultures, the 60-min exposure represents an intermediate timepoint, and the 120-min represents an extreme exposure time. The cells were washed to remove the release buffer, returned to culture buffer, cultured for 3 additional days, then analyzed. The results show that the release buffer exposure had a negligible effect on the cell viability after 3 days of further culture **(** **Figure 4B** **)**.

## 4 Discussion

The results presented here demonstrate the potential of using NK Cloudz microspheres to achieve effective feeder-cell-free *ex vivo* expansion of NK cells. The NK Cloudz results approached the results reported with K562-mbIL21-41BBL feeder cells: with a similar purity and expansion when cultured in the same protocol (**Supplementary Figure 2**), and a similar purity but lower expansion when the low-touch Cloudz/G-Rex6M protocol was compared with the high-touch feeder cell protocol (**Figure 3**). In addition, The Cloudz release process did not negatively affect the cell viability after a further 3 days in culture (**Figure 4**).

The expansion using NK Cloudz showed similar trends to previous feeder-based expansion of a clinical dose of NK cells in the larger G-Rex100 platform. Lapteva et al. examined NK cell expansion from PBMCs in a G-Rex100 with 400 ml of SCGM, 10% FBS, 10 U/ml IL-2 media along with K562-mbIL15-41BBL feeder cells. Lapteva et al. reported a mean purity of 70% ± 11% on day 8 and a mean fold expansion of 442 ± 29-fold on day 10 in 5 donors (15). In the smaller G-Rex6M well (100mL), we report 70% ± 11% purity and 300 ± 57-fold expansion on day 10 in the GMP SCGM, 10% FBS, IL-2/12/18/21 protocol in 4 donors. Literature suggests that the G-Rex platform is directly scalable up to larger vessels (40), and these results are comparable despite differences in the activator, cytokines, donors, and culture vessel size.

Importantly, when FBS was replaced with GMP Human Platelet Lysate in the developed protocol with Cloudz, the platelet lysate resulted in a similar NK purity and an 87-fold increase in the mean NK expansion on day 10 (**Figure 2**). These results align with previous reports that human platelet lysate performs equivalent or better than FBS in stem cell culture (41, 42).

The 387 ± 100-fold expansion of NK cells on day 10 when using the Cloudz and human platelet lysate (**Figure 2B**) protocols is significantly higher than the approximately 5-fold expansion reported on day 10 in 5% human platelet lysate, 1,000 IU/ml IL-2, and magnetic beads protocol reported by Huang et al. (25). Huang et al. reported 2,000-fold expansion by day 28 and the capacity for nonviral gene editing of the expanded population (25). The results here suggest that the NK Cloudz when combined with the G-Rex6M, GMP SCGM Media, GMP Human Platelet Lysate, and IL-2/12/18/21 results in a more rapid expansion of NK cells compared with the Huang et al. feeder-cell-free protocol. Our results also validate GMP Human Platelet Lysate as an equivalent xeno-free substitute for FBS in terms of NK purity and expansion on day 10.

Quintarelli et al. demonstrated a successful feeder-cell-free and FBS-free method to purify and expand NK cells using the MACS platform and 500 U/ml IL-2. The method achieved approximately 10-fold expansion by day 6 and 6,800-fold expansion by day 30. Quintarelli et al. further demonstrated that the expanded cells could be transduced with CAR.CD19 which both improved cytotoxicity against primary B-cell precursor acute lymphoblastic leukemia (Bcp-ALL) blasts and significantly reduced the tumor burden in mice, increasing their survival (27). Interestingly, MFI analysis of our phenotype markers showed similar trends to those reported by Quintarelli et al.: NKp46, CD2, and NKG2D increased in brightness, while CD16 expression dimmed (**Supplementary Figure 5**).

The K562-mbIL21-41BBL feeder cell approach has become the standard for *in vitro* NK expansion since publication of the Denman et al. manuscript in 2012. Denman et al. reported approximately 10-fold expansion on day 7, and greater than 1,000-fold expansion on day 14 using the K562-mbIL21-41BBL feeder cells cultured T75 flasks with RPMI-1640, 10% FBS, and 50 IU/ml IL-2 (6). In an effort to repeat this protocol, we measured 671-fold expansion on day 10; results that fit within the growth trajectory reported by Denman et al. (6) and more recently by Moseman et al. (36). Moseman et al. also found that AIM V media supplemented with an immune cell serum replacement (FBS-Free) was capable of more than twice the fold expansion compared with the RPMI/FBS protocol when starting with purified NK cells (36). However, our experiments to expand NK cells from PBMCs using AIM V and a serum replacement failed to produce significant expansion or purity (data not shown). When we compared the Cloudz/G-Rex6M protocol with the Denman et al. protocol, the Cloudz/G-Rex6M protocol resulted in a similar purity, viability, and cytotoxicity, but nearly half the fold expansion (**Figure 3**). The differences in expansion appear to be most closely associated with the protocol. When feeder cells were cultured using the same protocol as the Cloudz, the feeder cells resulted in a similar expansion, purity, viability, and cytotoxicity (**Supplementary Figure 2**). These results show a Cloudz-based feeder-cell-free and FBS-free approach that is capable of similar expansion, purity, and cytotoxicity compared with feeder cells.

The results raise a related observation associated with the protocols: The “low-touch” G-Rex6M protocol developed here does not require inputs for 10 days, which is beneficial for manufacturing. The flask-based feeder protocol was designed as a “high-touch” protocol with handling steps required every 2–3 days (**Figure 3A**). The higher-touch protocol produces nearly twice the mean fold expansion (despite large error bars), which suggests a trade-off between the number of labor-intensive handling steps in the protocol and higher expansion numbers.

We found that day 10 is the is the optimal culture time for the low-touch protocol in the G-Rex6M, presumably because the cells have reached the capacity of the well. The viability and cell population decrease after day 10 unless the cells are released and used (**Supplementary Figure 3** and **Figure 4**). For extended culture times, the protocol was modified to include more touchpoints to strategically increase the surface area and media available to the cells: the culture started in a G-Rex24 (8 ml), was transferred to a G-Rex6M on day 7, then split on days 10 and 14. Periodically splitting the cultures was important for continued growth. Using this higher-touch protocol, the Cloudz/G-Rex expansion was extended to day 20. The higher-touch Cloudz/G-Rex protocol achieved 55,106 ± 24,029-fold expansion, 96% ± 2% NK purity, and 88% ± 3% viability on day 20 (**Supplementary Figure 3**). These results are similar to the 47,967-fold expansion in 21 days reported in the K562-mbIL21-41BBL feeder cell protocol (6) and are greater than any known feeder-cell-free approach to date. The Cloudz and G-Rex maintained high NK expansion, purity, and viability over 20 days, and these results further show that the protocol is an important consideration for longer-term culture

The “high-touch” feeder cell protocol requires splitting of the cells on day 7, so the number of cells achieved based on the fold change calculation would require multiple flasks in parallel. The G-Rex6M protocol (198 × 10^6^ viable NK cells) supported 4.3 times more NK cells compared with the flask (46 × 10^6^ viable NK cells). A clinical dose was reported to be 20–50 × 10^6^ NK cells/kg (2, 6, 15), so an estimated expansion yield to support a clinical dose is 10 × 10^9^ NK cells (15). While it is theoretically possible to maintain enough parallel cultures to reach a clinical dose, it would require tens of G-Rex6M wells or hundreds of flasks. Both parallel experiments are impractical, which suggests further work is needed to scale efficient NK cell culture protocols to meet a clinically relevant dose. Wilson Wolf reports that the G-Rex6M can support 200–400 million cells, and our average culture expands to 273 million cells on day 10. So, in order to meet a clinical dose, future work will pursue methods to scale up to larger G-Rex culture vessels. The advantage of the G-Rex platform is that it is scalable up to larger volumes for clinical scale manufacturing, with the G-Rex100 (450 ml) process described by Lapteva et al. able to achieve a clinical dose (15). The extended Cloudz/G-Rex protocol (**Supplementary Figure 3**) is a step toward this progressive scale-up into larger G-Rex vessels over the course of the expansion to reach a clinical dose. Future work will focus on simplifying and further scaling the feeder-free NK manufacturing approach to reach a clinical dose using the feeder-free Cloudz-based method.

## Conclusions

The developed protocol utilizes NK Cloudz and GMP human platelet lysate to effectively expand cytotoxic NK cells 387-fold from a starting population of PBMCs. The protocol requires minimal interventions during the 10-day culture and relies on G-Rex6M scalable culture ware to deliver 198 million NK cells per well—with the potential to be scaled to meet clinical demands.

## Data Availability Statement

The original contributions presented in the study are included in the article/**Supplementary Material**. Further inquiries can be directed to the corresponding author.

## Author Contributions

CJ, NZ, EF, and JL designed the experiments. NZ and EF performed the experiments. CJ wrote the manuscript. All authors edited the manuscript. All authors listed have made a substantial, direct, and intellectual contribution to the work and approved it for publication.

## Funding

Funding was provided by Bio-Techne.

## Conflict of Interest

All authors are employees at Bio-Techne.

## Publisher’s Note

All claims expressed in this article are solely those of the authors and do not necessarily represent those of their affiliated organizations, or those of the publisher, the editors and the reviewers. Any product that may be evaluated in this article, or claim that may be made by its manufacturer, is not guaranteed or endorsed by the publisher.
