# Supplementary material for: Feeder-Cell-Free and Serum-Free Expansion of Natural Killer Cells Using Cloudz Microspheres, G-Rex6M, and Human Platelet Lysate
Source: Front Immunol. 2022 Mar 7;13:803380. doi: 10.3389/fimmu.2022.803380 (PMC8934851; doi:10.3389/fimmu.2022.803380)
Supplement: Supplementary file 1 [file Presentation_1.pdf]

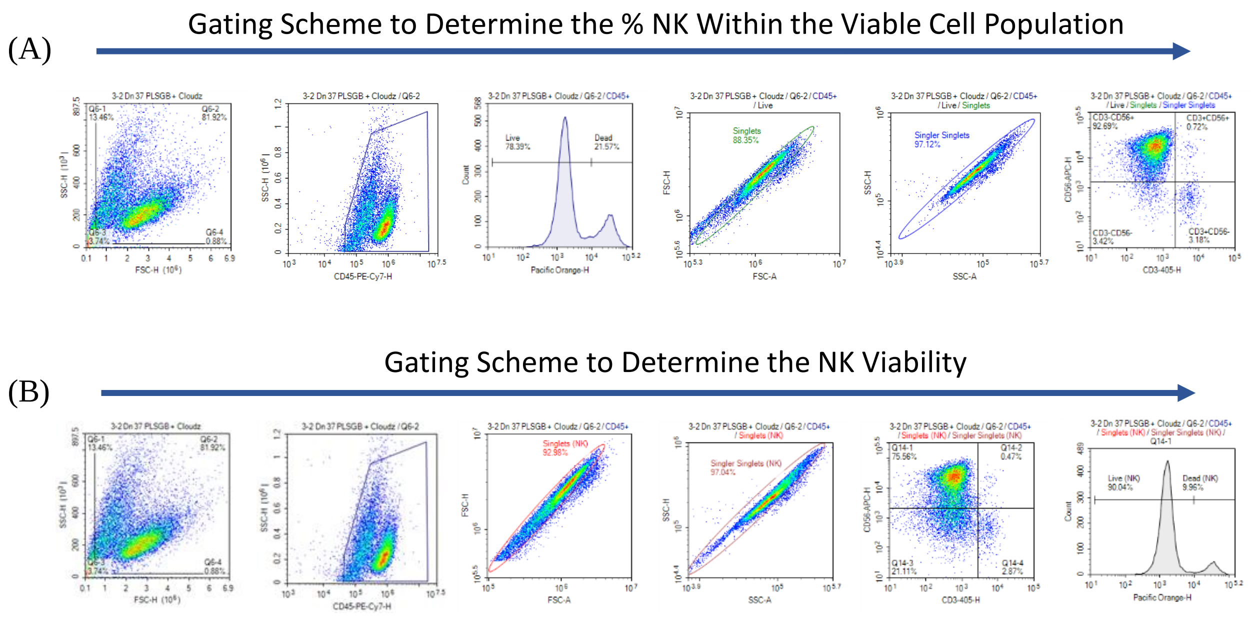


**Supplementary Figure 1. Gating scheme employed to determine (A) the cell distribution and (B) the NK viability**. Our strategy was to include the smaller events in the initial gate in order to be conservative in our viability reporting. For both methods: a broad size/granularity gate ensured that a majority of the events were carried forward. A CD45 gate, based on unstained samples, was applied to distinguish cells from non-cells. For **(A)** the cell distribution, a viability gate was applied followed by FSC and SSC singlets to remove clumps of cells. The CD3/CD56 cell distribution was then reported for viable CD45+ single events. To determine the NK viability, the process used the same gates as **(A)**, but the viability gate was applied last and was based only on the CD45+CD3-CD56+ singlets population.

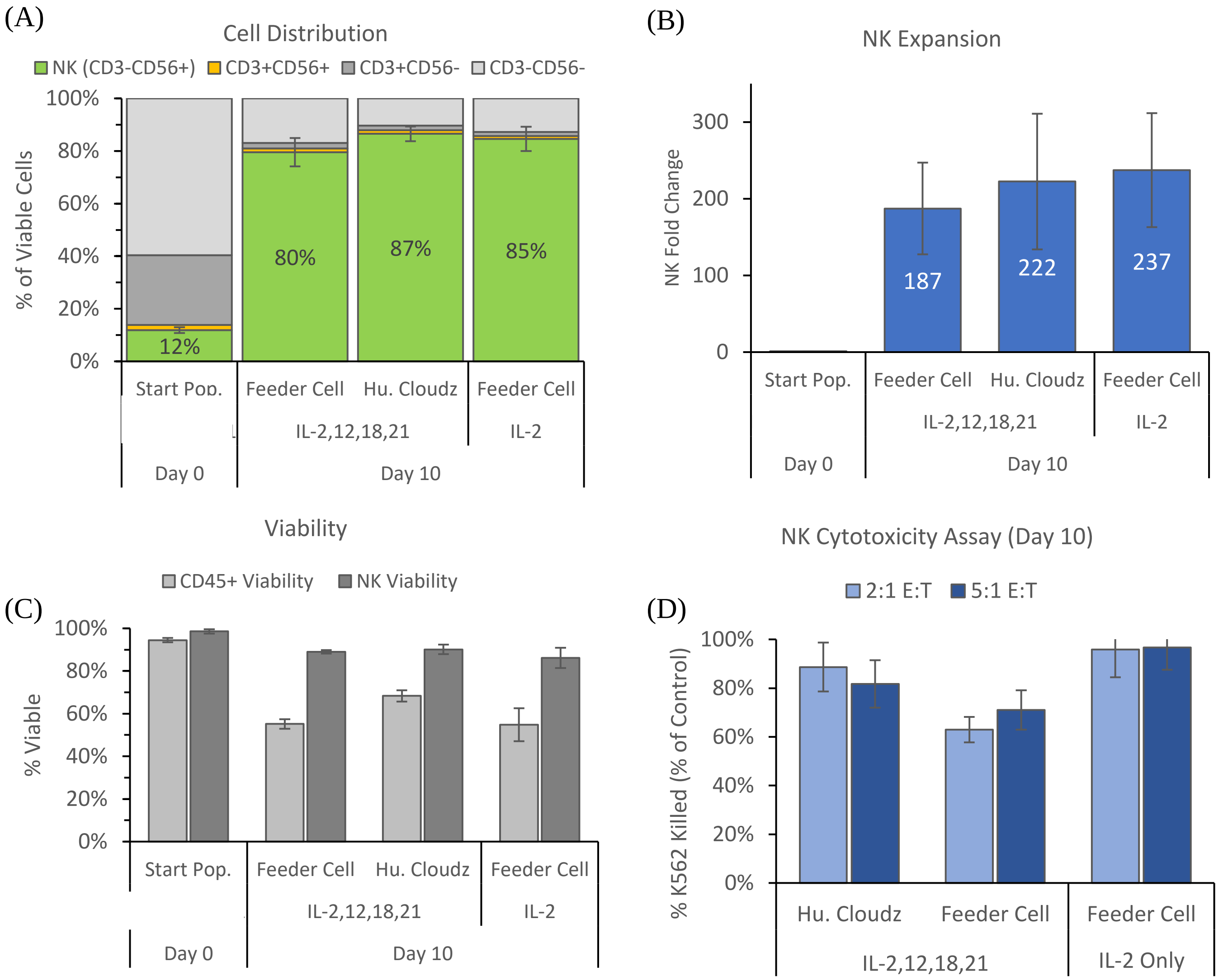


**Supplementary Figure 2. Exchange of Cloudz for K562-mbIL21-41BBL feeder cells in the same protocol.** These experiments were performed in the SCGM + 10% FBS with IL-2/12/18/21 in a T25 flask protocol where ½ media exchange was performed every 3 days. The protocol was modified from Denman et al. for Cloudz culture in flasks. Experiments were performed by replacing the Cloudz with K562-mbIL21-41BBL feeder cells in the flasks with either our cytokine scheme (IL-2/12/18/21) or the feeder cell cytokine scheme (50 IU/mL IL-2). The only other modification was that the feeder cells were seeded at a 2:1 Feeder:NK ratio and were replenished at a 1:1 ratio on day 7. (based on Denman et al.) The Cloudz were seeded at 75µL/500,000NK cells and were not replenished for the duration of the experiment. The results show **(A)** the cell distribution on days 0 and 10, **(B)** NK (CD3-CD56+) fold-change between day 0 and day 10, **(C)** the viability of either all CD45+ cells or NK cells alone on Days 0 or 10, and **(D)** the percent of target K562 cells killed on day 10 with either a 2:1 or 5:1 effector:target (E:T) ratio and a 4-hour incubation. Data represents the mean ± S.D. from 3 separate donors. When Cloudz and feeder cells are compared in the same protocol, the mean purity, expansion, and viability were similar. The mean cytotoxicity decreased relative to the Cloudz. When the feeder cells were cultured with IL-2 alone, the mean purity, expansion, and viability again remained similar, and the cytotoxicity increased to greater than 95%. These results suggest that when the same protocol was applied, the expansion between Cloudz and feeder cells was similar **(B)**.

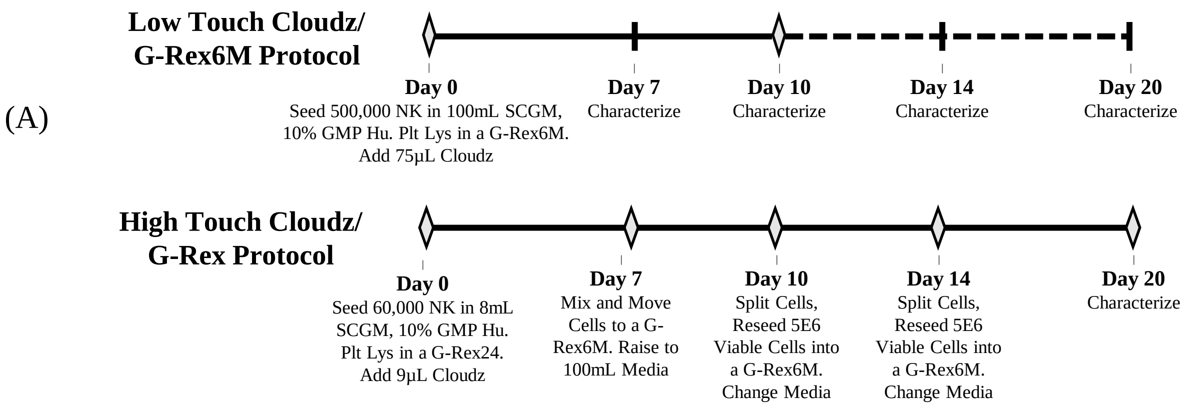


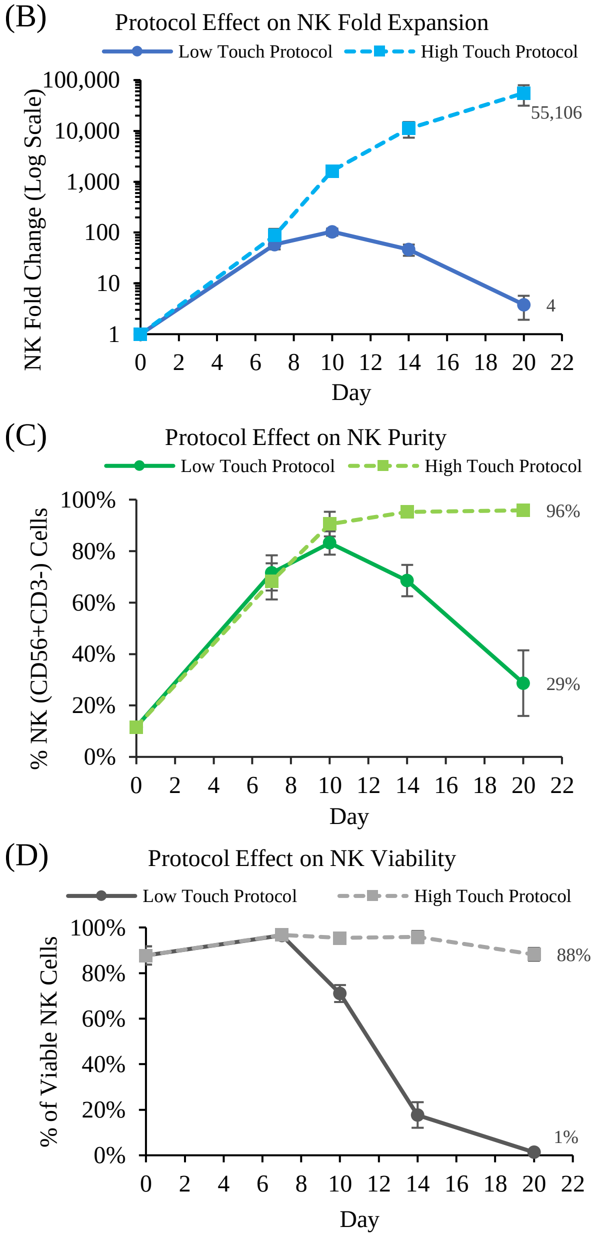


**Supplementary Figure 3. A high-touch Cloudz/G-Rex protocol modification for extended culture periods. (A)** The existing low-touch protocol was extended to 20-days and compared to a revised high-touch protocol that starts in 8mL in a G-Rex24 and is progressively transferred or split over time. **(B)** Compares the NK fold-expansion **(C)** Compares the NK purity, and **(D)** compares NK viability between the low-touch protocol (solid lines) and the high-touch Cloudz/G-Rex protocol (dotted-lines). The high-touch Cloudz/G-Rex protocol delivered consistent expansion over 20-days, reaching 55,106±24,029-fold expansion, 96±3% purity, and 88±3% viability of NK cells by day 20. The low-touch protocol, which received no media exchanges, peaked on day 10 and crashed by day 20. Our previous experiments have shown that media exchanges can rescue the purity and viability to some extent, but not the expansion (data not shown). This suggests that splitting the cultures is an important variable for maintain growth in the NK cultures. Data represents the mean ± standard deviation from 3 separate donors.

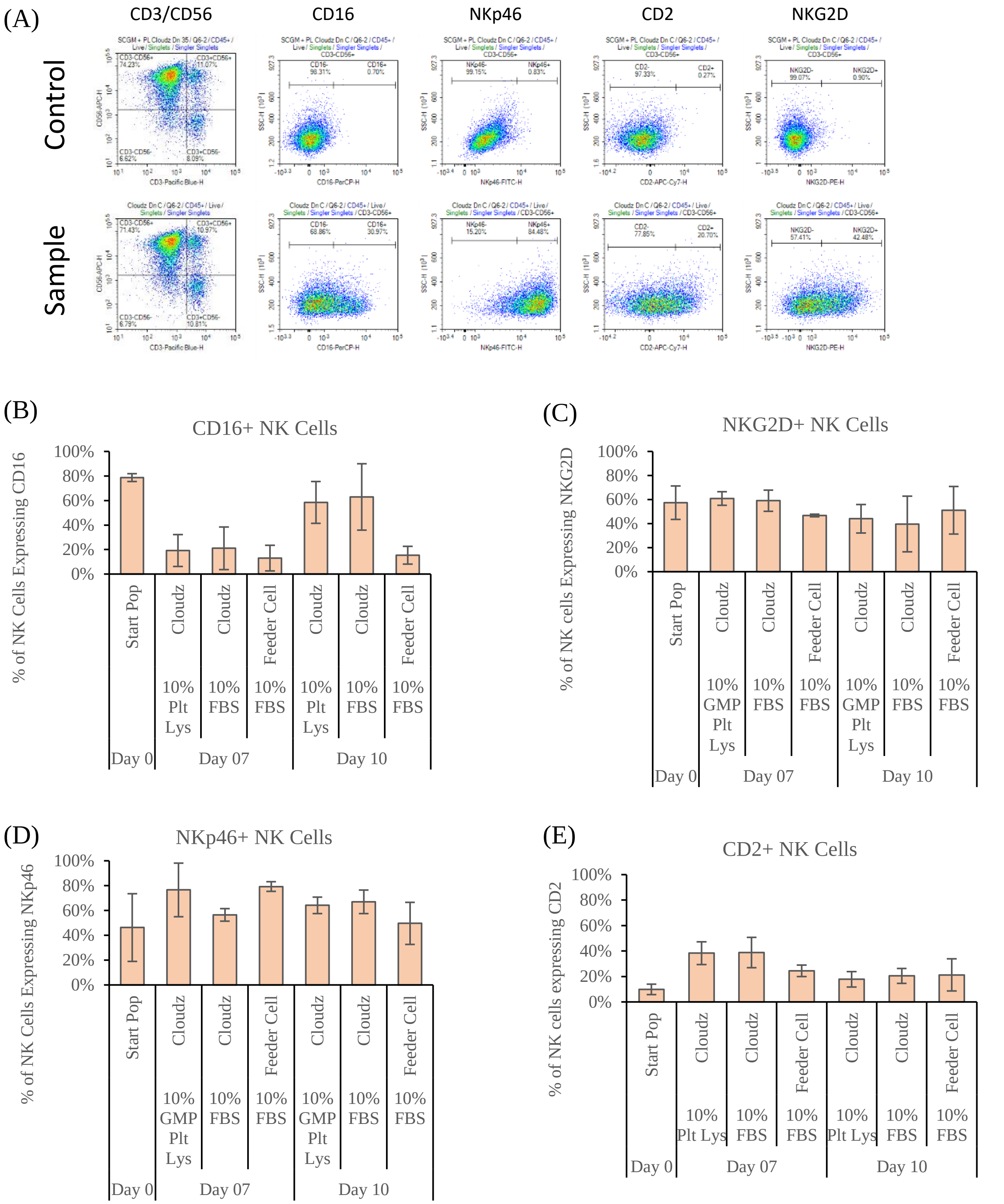


**Supplementary Figure 4. Phenotype analysis of activating ligand expression under different culture conditions.** Cells were cultured with the Cloudz/G-Rex protocol using either FBS or GMP human platelet lysate, or with the feeder cell/flask protocol. **(A)** shows representative gating based on a matched control stained with CD3, CD45, and CD56. The results show **(B)** the percentage of cells expressing CD16 on days 0,7,and 10, **(C)** the percentage of cells expressing NKG2D on days 0,7,and 10, **(D)** the percentage of cells expressing NKp46 on days 0,7,and 10, and **(E)** the percentage of cells expressing CD2 on days 0,7,and 10. The results represent the mean ± standard deviation from 3 separate donors.

(A)

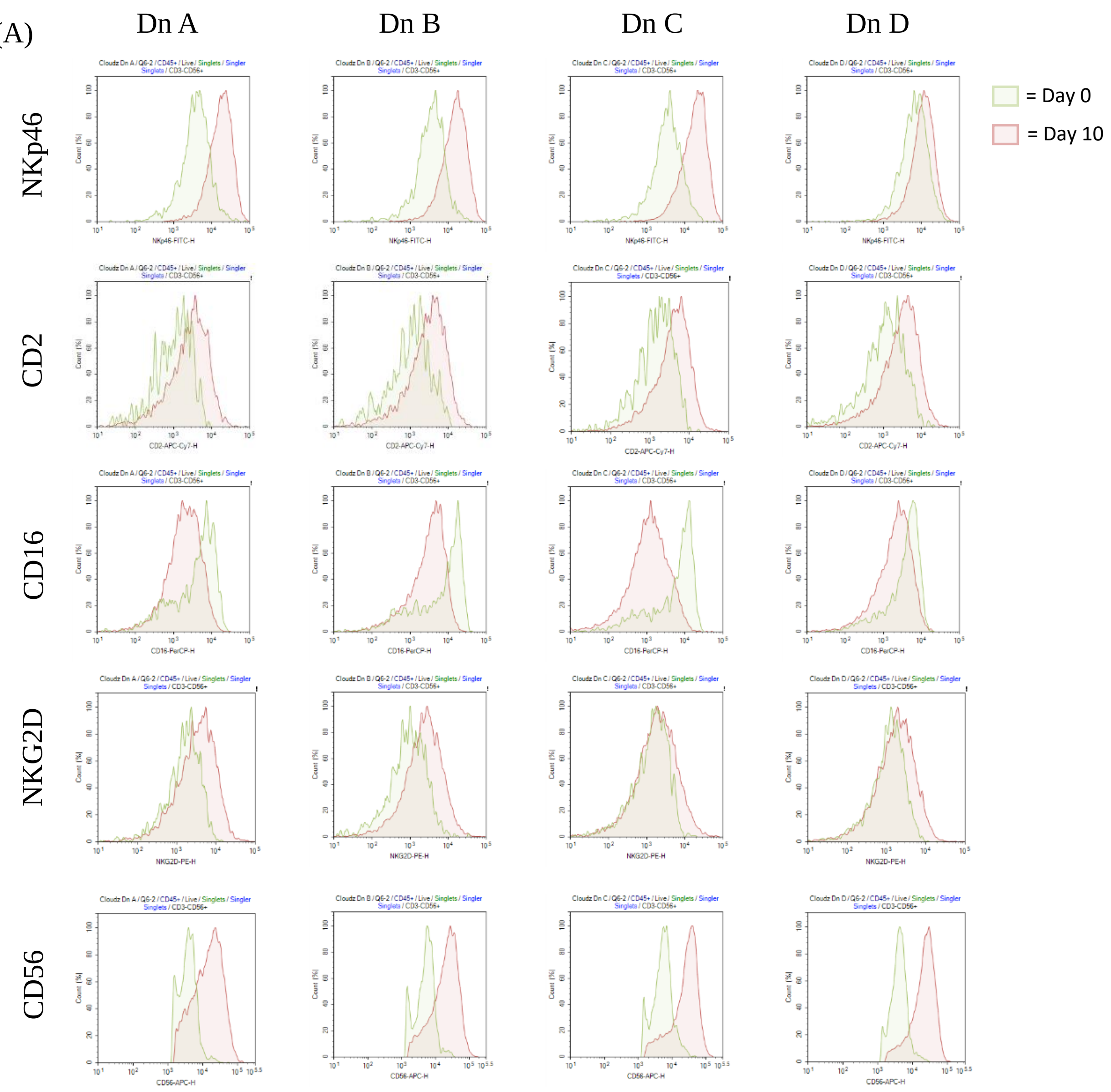


(B)

| | | Day 7 | | | Day 10 | | | Day 14 | | |
|---|---|---|---|---|---|---|---|---|---|---|
| | | Fold Change | % NK | % Viable Cells | Fold Change | % NK | % Viable Cells | Fold Change | % NK | % Viable Cells |
| Day 7 | NKp46+ MFI | 0.56 | 0.45 | -0.62 | 0.46 | -0.09 | 0.22 | 0.77 | 0.64 | 0.83 |
| | CD2+ MFI | -0.11 | 0.47 | 0.59 | -0.02 | 0.62 | -0.52 | -0.47 | 0.30 | -0.39 |
| | CD56+ MFI | 0.15 | 0.31 | -0.22 | 0.38 | 0.30 | -0.30 | 0.13 | 0.18 | -0.06 |
| | NKG2D+ MFI | 0.21 | 0.09 | -0.57 | 0.29 | -0.22 | 0.51 | 0.71 | 0.28 | 0.69 |
| | CD16+ MFI | -0.40 | -0.42 | 0.44 | -0.35 | -0.20 | -0.13 | -0.33 | -0.53 | -0.40 |
| Day 10 | NKp46+ MFI | -0.02 | -0.40 | -0.69 | -0.05 | -0.58 | 0.64 | 0.46 | -0.14 | 0.48 |
| | CD2+ MFI | 0.73 | 0.40 | -0.93 | 0.75 | -0.14 | 0.24 | 0.87 | 0.46 | 0.80 |
| | CD56+ MFI | 0.29 | 0.46 | -0.57 | 0.47 | 0.27 | 0.01 | 0.43 | 0.49 | 0.31 |
| | NKG2D+ MFI | 0.66 | 0.25 | -0.90 | 0.60 | -0.32 | 0.47 | 0.97 | 0.40 | 0.94 |
| | CD16+ MFI | -0.51 | -0.35 | 0.38 | -0.54 | -0.04 | -0.28 | -0.30 | -0.25 | -0.44 |
| Day 14 | NKp46+ MFI | 0.55 | 0.22 | -0.97 | 0.54 | -0.27 | 0.51 | 0.81 | 0.40 | 0.83 |
| | CD2+ MFI | -0.28 | 0.23 | 0.78 | -0.83 | -0.66 | -0.57 | -0.75 | 0.03 | -0.69 |
| | CD56+ MFI | 0.68 | 0.59 | -0.76 | 0.76 | 0.10 | 0.00 | 0.76 | 0.58 | 0.64 |
| | NKG2D+ MFI | 0.69 | 0.29 | -0.94 | 0.67 | -0.26 | 0.40 | 0.94 | 0.42 | 0.89 |
| | CD16+ MFI | -0.62 | -0.31 | 0.67 | -0.62 | 0.16 | -0.40 | -0.56 | -0.31 | -0.68 |

**Supplementary Figure 5. Mean fluorescent intensity of NKp46, CD2, CD16 and NKG2D and correlation with culture outcomes**. **(A)** MFI Histograms based on viable CD45+CD3-CD56+ cell populations from 4 donors cultured using the G-Rex6M/Cloudz/GMP Plt Lys protocol. The green plots show the distribution of intensities on Day 0 and the red plots show the distribution of intensities on Day 10. NKp46, CD2, NKG2D and CD56 intensity increased in the expanded NK cells populations on day 10, while CD16 MFI decreased.

**Supplementary Figure 5 continued. (B) A table of coefficients assessing correlation between MFI and experimental outcomes.** The Pearson correlation coefficient provides a number ranging from -1 to 1 to represent the degree of correlation between two paired sets of data. A value of 1 describes two data sets that are perfectly correlated,  while -1 indicates a perfect inverse correlation. Coefficients are generally considered high if they are greater than 0.7 or less than -0.7 (Mukaka). Here, the correlation coefficients were organized into a table to provide a heat map of high and low correlation between groups in order to identify consistent correlations associated with certain markers. Each coefficient includes values from the SCGM + 10% FBS + Cloudz, SCGM + 10% Plt Lys + Cloudz, and RPMI + 10% FBS + Feeders conditions.

Mukaka M. A guide to appropriate use of Correlation coefficient in medical research. *Malawi Med J* (2012) 24:69–71.

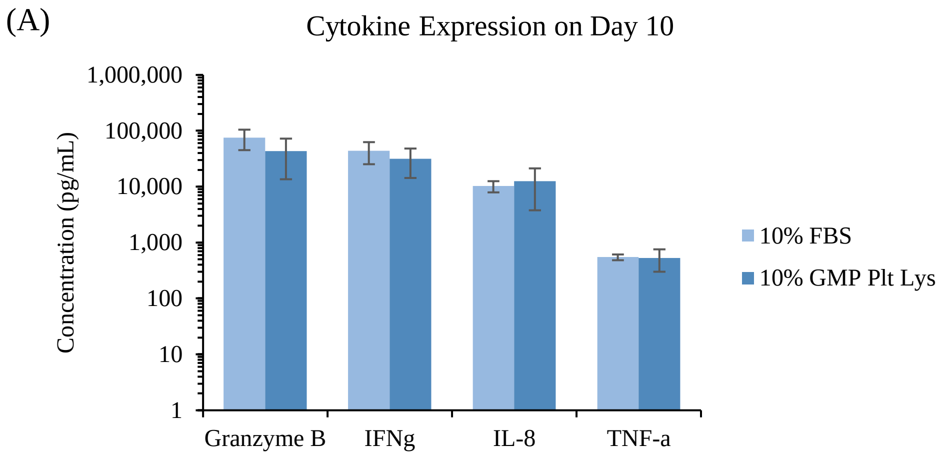


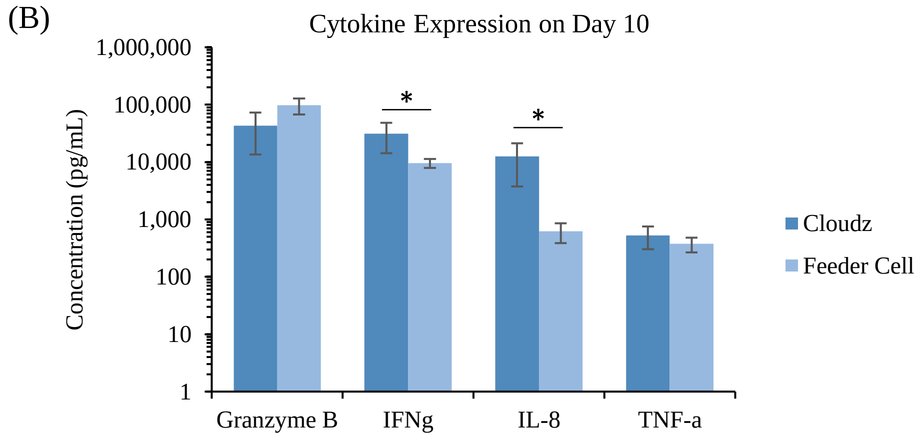


**Supplementary Figure 6. ELLA analysis of cytokine concentration in the media on day 10.** The ELLA is an automated microfluidic method for performing an ELISA on multiple analytes simultaneously. **(A)** represents the mean concentration (pg/mL) of Granzyme B, IFNg, IL-8 and TNF-a in the media in the G-Rex6M/Cloudz protocol supplemented with either 10% FBS or 10% GMP human platelet lysate. **(B)** represents the mean concentration (pg/mL) of Granzyme B, IFNg, IL-8 and TNF-a in the media in either the Cloudz/G-Rex6M/GMP human platelet lysate protocol or the feeder cell/flask protocol. Data represents the mean ± standard deviation from 3 separate donors.

Methods: On day 10 of culture 1mL samples from each sample well were transferred to a sterile 1.5mL sterile tube and frozen at -20°C until use. On the day of use, samples were thawed in a 37°C waterbath, then briefly vortexed to mix. Samples were then centrifuged at 8000 x G for 5 minutes to pellet precipitates. Samples were then diluted 1:100 in ELLA running buffer. Samples were loaded into cartridges (SPCKC-CS-005511, ProteinSimple) equipped to multiplex Granzyme B, Interferon Gamma (IFNg), IL-8, and Tumor Necrosis Factor Alpha (TNF-a) and run according to the manufacturer’s instructions.
